# Herpes Simplex Virus ICP27 Protein Inhibits AIM 2-Dependent Inflammasome Influencing Pro-Inflammatory Cytokines Release in Human Pigment Epithelial Cells (hTert-RPE 1)

**DOI:** 10.3390/ijms25094608

**Published:** 2024-04-23

**Authors:** Anna Caproni, Chiara Nordi, Riccardo Fontana, Martina Facchini, Sara Melija, Mariangela Pappadà, Mattia Buratto, Peggy Marconi

**Affiliations:** 1 Department of Chemical, Pharmaceutical and Agricultural Sciences, University of Ferrara, 44121 Ferrara, Italy; anna.caproni@unife.it (A.C.); chiara.nordi@unife.it (C.N.); fntrcr1@unife.it (R.F.); martina.facchini@unife.it (M.F.); sara.melija@unife.it (S.M.); mariangela.pappada@unife.it (M.P.); mattia.buratto@unife.it (M.B.); 2 LTTA Laboratory for Advanced Therapies, Technopole of Ferrara, 44121 Ferrara, Italy

**Keywords:** viruses, host–virus interaction, innate immunity, inflammasome

## Abstract

Although Herpes simplex virus type 1 (HSV-1) has been deeply studied, significant gaps remain in the fundamental understanding of HSV-host interactions: our work focused on studying the Infected Cell Protein 27 (ICP27) as an inhibitor of the Absent-in-melanoma-2 (AIM 2) inflammasome pathway, leading to reduced pro-inflammatory cytokines that influence the activation of a protective innate immune response to infection. To assess the inhibition of the inflammasome by the ICP27, hTert-immortalized Retinal Pigment Epithelial cells (hTert-RPE 1) infected with HSV-1 wild type were compared to HSV-1 lacking functional ICP27 (HSV-1∆ICP27) infected cells. The activation of the inflammasome by HSV-1∆ICP27 was demonstrated by quantifying the gene and protein expression of the inflammasome constituents using real-time PCR and Western blot. The detection of the cleavage of the pro-caspase-1 into the active form was performed by using a bioluminescent assay, while the quantification of interleukins 1β (IL-1β) and 18 (IL-18)released in the supernatant was quantified using an ELISA assay. The data showed that the presence of the ICP27 expressed by HSV-1 induces, in contrast to HSV-1∆ICP27 vector, a significant downregulation of AIM 2 inflammasome constituent proteins and, consequently, the release of pro-inflammatory interleukins into the extracellular environment reducing an effective response in counteracting infection.

## 1. Introduction

Herpes simplex virus type 1 (HSV-1) is a neurotropic human pathogen that establishes a long-term interaction with the infected host. Primary infection is followed by an ascension in the axons of the sensory nerve, which results in a latent infection in the ganglia [1]. The reactivation of latent virus infection is a phenomenon affecting most patients with HSV-1 and is strictly associated with the host immune status [2]. Recurring active infections, manifesting as either symptomatic or asymptomatic cases (thus potentially spreading unknowingly), contribute to various clinical conditions, such as cold sores, keratitis, blepharitis, meningitis, encephalitis, and genital infections, which can lead to significant complications in neonatal and immunocompromised individuals [3,4]. The herpetic sores observed during initial infection are closely related to the immune system: the recognition of HSV-1 by pattern recognition receptors (PRRs) initiates the expression of cytokines that play a key role in determining the resolution of the disease within 7 to 14 days [5,6,7].

One of the main signaling systems of the innate immune response is the intracellular formation of inflammasomes. They are multi-protein cytoplasmic complexes responsible for producing inflammatory cytokines, such as interleukin 1β (IL-1β) and interleukin 18 (IL-18), as well as inflammatory programmed cell death called pyroptosis [8,9]. Most of the known inflammasomes act through the activation of the enzyme caspase-1 and are called “canonical inflammasomes”; however, some studies have also described “non-canonical inflammasomes” that act through the activation of caspases 4 and 5, which play the same role as caspase-1 [10]. Canonical inflammasomes are structurally different but have three main components: sensor, adaptor protein, and enzyme precursor [11]. The sensor is the initiating protein that recognizes the pathogen- and damage-associated molecular patterns (PAMPs and DAMPs): indeed, there are several families of inflammasome sensors such as NOD-like receptors (NLRs), AIM 2-like receptors (ALRs), and RIG-I-like receptors (RLRs) [12]. Inflammasome sensor proteins (NLRs, ALRs, and RLRs) detect signals at the cytoplasmic or nuclear level [13]. The recognition of PAMPs and DAMPs induces the sensor proteins to oligomerize, binding to each other with homotypic interactions between shared domains, and recruit the adaptor protein apoptosis-associated speck-like protein containing a caspase recruitment domain (CARD) (ASC). The binding of the sensor protein to the ASC adaptor induces a conformational change of other ASC molecules present in the cytosol: this leads to the formation of ASC filaments that cluster and recruit the inactive enzyme precursor pro-caspase-1. In this way, pro-caspase-1 undergoes self-cleavage to produce active caspase-1 that leads to the activation of specific pro-inflammatory cytokines: IL-1β and IL-18 [14,15,16]. IL-1β has a potent pyrogenic effect: it activates immune cells and promotes the increase in adhesion molecules on endothelial cells to allow the migration of activated immune cells to the site of infection [17]. IL-18 is the second cytokine processed in its bio-active form by the enzyme caspase-1 activated by canonical inflammasomes and, like IL-1β, binding to its receptor activates the transcription factors NF-kB (nuclear factor kappa-light-chain-enhancer of activated B cells), p38 (mitogen-activated protein kinases), and JNK (c-Jun NH2-terminal kinase) [8,18,19]. 

**Figure 1:**
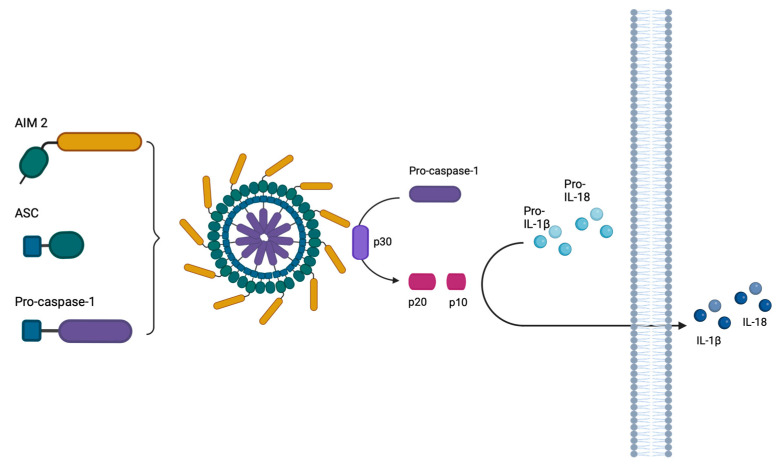
The oligomerization of AIM 2, ASC, and pro-caspase-1 proteins results in the formation of the heptosome after the AIM 2 sensor recognizes viral DNA that has been released into the cytoplasm. Proteins within the inflammasome aggregate, causing pro-caspase-1 to be processed into its temporary form, p30. The CARD domain (p10) is removed during self-cleavage of p30, releasing the active caspase p20. Interleukins 1β and 18 are converted into their active form by active caspase-1 and released into the extracellular milieu. The figure was generated using Biorender.com.

During HSV-1 infection, the NLRP3 (NLR family pyrin domain containing 3), AIM 2 (Absent-in-melanoma-2), IFI16 (Interferon-inducible protein 16), and RIG-1 (Retinoic acid-inducible gene I)-dependent inflammasomes are known to be activated [20,21,22,23,24,25,26,27,28]. Specifically, AIM 2 is a sensor that can detect double-stranded DNA at the cytoplasmic level through its hematopoietic interferon-inducible nuclear HIN (hematopoietic interferon-inducible nuclear domain): its activation leads to the formation of the AIM 2 inflammasome through the oligomerization of AIM 2 protein sensor with ASC and pro-caspase-1, resulting in the activation of caspase-1 (p30 auto-clivated to p20 and p10) and, consequently, the release of IL-1β and IL-18 (Figure 1) [29,30,31,32,33]. 

Horan et al. explained how the viral DNA of HSV-1 is released at the cytoplasmic level via the proteolytic cleavage of the viral nucleocapsid by the host cell’s ubiquitin protease: the viral DNA released into the cytoplasm acts, therefore, as a substrate for the AIM 2 protein that triggers the inflammasome formation [34,35]. Other studies, however, showed that HSV-1 is able to activate the inflammasome in macrophages but without the activation of AIM 2, contrary to what is observed in keratinocytes, suggesting different pathways depending on the cell types [36,37]. Therefore, HSV-1 may have evolved distinct mechanisms to evade AIM 2-dependent inflammasome activation: it has been observed that the HSV-1 tegument protein VP22 prevents AIM 2-dependent inflammasome activation, thereby attenuating interleukin secretion in macrophages upon infection [38,39]. Based on these assumptions, the role of some HSV-1 genes in immune evasion needs to be investigated in more detail [40].

Among HSV-1 proteins, ICP27 (Infected cell protein 27) is a multifunctional protein encoded by gene *UL54* (Unique Long region 54) that plays essential roles in viral infection: it is involved in the modulation of viral gene expression, exerting control over key processes such as transcription and translation; in RNA splicing, influencing the maturation of viral transcripts; and, finally, it is involved in facilitating the nuclear export of viral RNA, thus contributing to the efficient assembly of infectious viral particles [41,42]. In addition to its direct impact on viral processes, Sandri-Goldin’s work highlights the broader influence of ICP27 on host cellular functions, such as the cell cycle, the activation of stress signaling pathways, and the prevention of apoptosis [42,43,44,45]. The regulatory protein ICP27 has been shown to interact with host cell factors, potentially affecting the expression and function of cellular genes [46,47,48]. Indeed, recent studies demonstrate how ICP27 is involved in key molecular mechanisms that allow HSV-1 to manipulate host cellular pathways to facilitate its replication and immune evasion [49,50,51,52]. This intricate interaction between ICP27 and host cellular components underscores the complexity and gaps of virus–host interaction during HSV-1 infection. Therefore, this work aimed to evaluate the role of ICP27 as an inhibitor of the AIM 2 inflammasome and, consequently, as an agent of immune evasion by reducing the release of pro-inflammatory interleukins necessary to induce an effective innate immune response. 

## 2. Results 

### 2.1. HSV-1ΔICP27 Mutant Virus Does Not Express the ICP27 Protein

**Figure 2:**
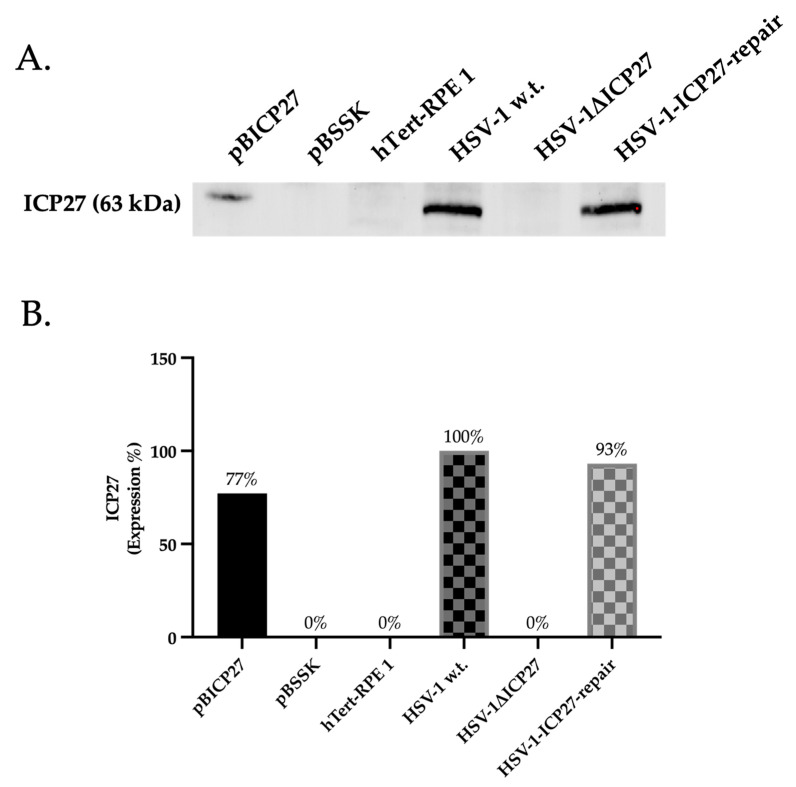
Evaluation of presence/absence of ICP27 protein. (**A**) Western blot assay for ICP27 protein in cells transfected with pBICP27 and pBSSK and in cells infected with HSV-1 w.t., HSV-1ΔICP27, and HSV-1-ICP27-repair at 24 h post-transfection or infection. (**B**) Densitometric quantification of ICP27 levels was performed using stain-free blotting, and the results were reported as a percentage calculated from the ratio of normalized protein levels to total protein (Supplementary Materials—Figures S1 and S2). Relative percentages correspond to the proportions of HSV-1 w.t. (100%) infected cells. Detection of total proteins and band was performed using the Chemidoc^TM^MP Imaging System (Biorad, Segrate (MI), Italy) and normalization was performed using Image Lab Software (6.0.0, Biorad, Segrate (MI), Italy).

In order to evaluate the effect of the HSV-1 ICP27 protein on the AIM 2 inflammasome pathway, we verified at multiple levels how the loss of the *ICP27* gene in the HSV-1ΔICP27 virus can alter the expression and biological activity of the signalosome. Thus, to ascertain whether or not the ICP27 protein was present, we first performed a Western blot analysis to demonstrate that the virus HSV-1ΔICP27 did not express the *ICP27* gene. Specifically, the presence or absence of *ICP27* was verified in Retinal Pigment Epithelial (hTert-RPE 1) cells transfected with plasmids, such as pBICP27, utilized for vector creation and its control, pBSSK, and cells infected with HSV-1 w.t. (wild type), HSV-1ΔICP27, and HSV-1-ICP27-repair. As a result (Figure 2), the ICP27 protein was present only in samples transfected with the plasmid expressing the *ICP27* gene (pBICP27) (77%) and in samples infected with HSV-1 w.t. (100%) and HSV-1-ICP27-repair (93%). Since the results obtained using HSV-1 w.t. and HSV-1-ICP27-repair are comparable, in this study the subsequent experiments shown include only cells untreated or infected with HSV-1 w.t. and HSV-1ΔICP27 virus.

### 2.2. HSV-1 ICP27 Protein Inhibits the Inflammasome’s Sensor Protein AIM 2

**Figure 3:**
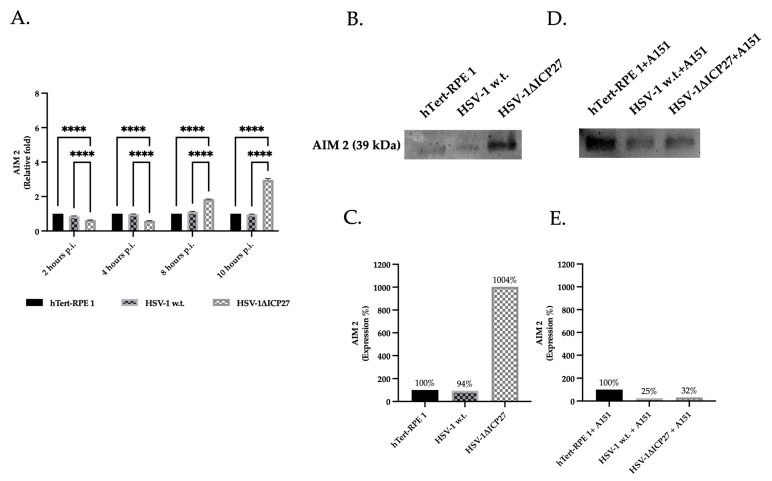
Gene and protein expression of AIM 2 sensor protein. (**A**) Real-time PCR of untreated hTert-RPE 1, HSV-1 w.t., and HSV-1ΔICP27-infected cells (M.O.I. of 3) at 2, 4, 8, and 10 h.p.i. Relative fold changes are related to the levels in untreated cells (hTert-RPE 1). Values are averages of SEM ± from three biological replicates. Two-way ANOVA was performed. ****, *p* < 0.0001. (**B**,**D**) Western blot assay for AIM 2 in both untreated (**B**) and pre-treated with AIM 2 inhibitor A151 (**D**) hTert-RPE 1, HSV-1 w.t., and HSV-1ΔICP27-infected cells (M.O.I. of 3) at 10 h.p.i. (**C**,**E**) Densitometric quantification of AIM 2 levels was performed using stain-free blotting, and the results were reported as a percentage calculated from the ratio of normalized protein levels to total protein (Supplementary Materials: Figures S3–S6). Relative percentages correspond to the proportions of untreated (100%) cells. Detection of both total protein (stain-free blot) and detected bands was performed using the Chemidoc^TM^MP Imaging System (Biorad) and normalization was performed using Image Lab Software (Biorad).

hTert-RPE 1 cells infected with 3 M.O.I of either HSV-1 w.t. or HSV-1ΔICP27 mutant virus were compared to evaluate the impact of ICP27 on the AIM 2 sensor protein. Untreated hTert-RPE 1 cells represented the negative control. Since ICP27 is actively synthesized by the HSV-1 w.t. virus approximately 4 h.p.i. (hours post-infection), real-time PCR was employed to quantify AIM 2 RNA levels at 2, 4, 8, and 10 h.p.i., in order to elucidate the impact of ICP27 during the early stages of infection (Figure 3A). The relative fold change of AIM 2 at 2 and 4 h.p.i. in cells infected with both HSV-1 w.t. and HSV-1ΔICP27 mutant virus appeared to be comparable or lower compared to untreated cells, respectively. Starting at 8 h.p.i., a reversal of this pattern was observed, manifesting as a significant increase in the relative fold change of AIM 2 in cells infected with the ICP27 deleted virus HSV-1ΔICP27. 

Additionally, the protein quantification of cells infected for 10 h shows that, in comparison to untreated and HSV-1ΔICP27 infected cells, the presence of the ICP27 protein in cells infected with HSV-1 w.t. caused a marked decrease in the AIM 2 protein. As seen in Figure 3B,C, the protein quantification of AIM 2 in HSV-1ΔICP27-infected cells was, in fact, consistently higher (1004%) than in untreated (100%) or HSV-1-infected cells (94%). Additionally, hTert-RPE 1 control cells and cells infected with HSV-1 w.t. or HSV-1ΔICP27 were pre-treated with the AIM 2 inhibitor ODN TTAGGG (A151) in order to validate the inhibition imposed by ICP27 (Figure 3D,E). The findings show that cells being pre-treated with AIM 2 inhibitor and infected with HSV-1ΔICP27 (32%) behave similarly to cells infected with HSV-1 w.t. (25%) compared to 100% of uninfected cells.

### 2.3. HSV-1’s ICP27 Protein Inhibits the Inflammasome’s Adaptor Protein ASC

**Figure 4:**
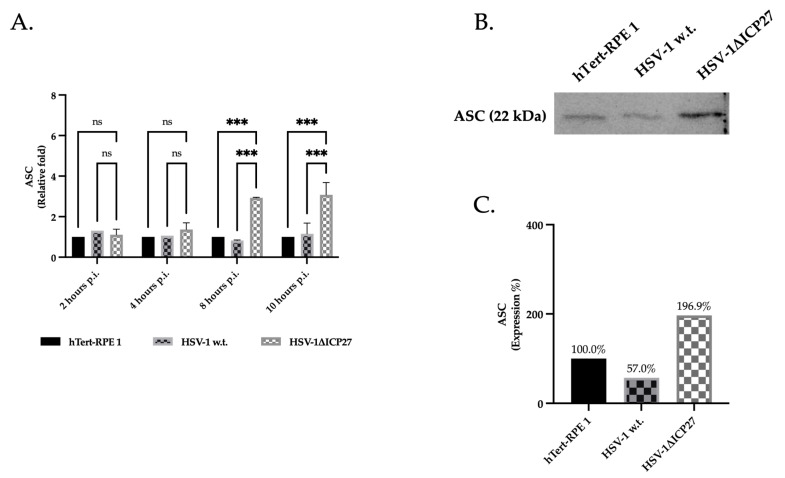
Gene and protein expression of ASC adaptor protein. (**A**) Real-time PCR of untreated hTert-RPE 1, HSV-1 w.t., and HSV-1-ΔICP27-infected cells (M.O.I. of 3) at 2, 4, 8, and 10 h.p.i. Relative fold changes are related to the levels in untreated cells (hTert-RPE 1). Values are averages SEM ± from three biological replicates. Two-way ANOVA was performed. ns *p* ≥ 0.1, ***, *p* < 0.001. (**B**) Western blot assay for ASC (22 kDa) in both untreated hTert-RPE 1, HSV-1 w.t., and HSV-1ΔICP27-infected cells (M.O.I. of 3) at 10 h.p.i. (**C**) Densitometric quantification of ASC levels was performed using stain-free blotting, and the results were reported as a percentage calculated from the ratio of normalized protein levels to total protein (Supplementary Materials—Figures S7 and S8). Relative percentages correspond to the proportions of untreated (100%) cells. Detection of both total protein (stain-free blot) and detected bands was performed using the Chemidoc^TM^MP Imaging System (Biorad) and normalization was performed using Image Lab Software (Biorad).

To assess whether HSV-1’s ICP27 protein can reduce ASC protein expression, we evaluated the RNA and protein levels of ASC in untreated cells, HSV-1 w.t., and HSV-1ΔICP27 mutant virus-infected cells (Figure 4A). The evaluation of *ASC* gene expression at 2, 4, 8, and 10 h post-infection revealed a notable rise in the relative fold of ASC protein beginning at 8 h post-infection. The Western blot analysis of protein expression in hTert-RPE 1 cells infected for 10 h confirmed the results of real-time PCR performed at 10 h.p.i. (Figure 4B). In summary, cells infected with the *ICP27* gene-deleted virus exhibit much greater protein production of ASC (196.9%) compared to 100% of untreated cells and HSV-1 w.t. infected cells (57%). 

### 2.4. HSV-1 ICP27 Protein Inhibits the Inflammasome’s Effector Protein Pro-Caspase-1

**Figure 5:**
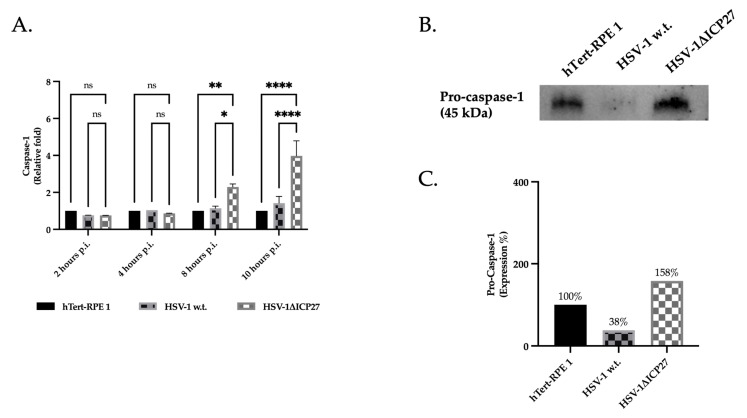
Gene and protein expression of pro-caspase-1. (**A**) Real-time PCR of untreated hTert-RPE 1, HSV-1 w.t., and HSV-1ΔICP27-infected cells (M.O.I. of 3) at 2, 4, 8, and 10 h.p.i. Relative fold changes are related to the levels in untreated cells (hTert-RPE 1). Values are averages SEM ± from three biological replicates. Two-way ANOVA was performed. ns *p* ≥ 0.1, * *p* < 0.1, ** *p* < 0.01, ****, *p* < 0.0001. (**B**) Western blot assay for pro-caspase-1 (45 kDa) in untreated hTert-RPE 1, HSV-1 w.t., and HSV-1ΔICP27-infected cells (M.O.I. of 3) at 10 h.p.i. (**C**) Densitometric quantification of pro-caspase-1 levels was performed using stain-free blotting, and the results were reported as a percentage calculated from the ratio of normalized protein levels to total protein (Supplementary Materials—Figures S9 and S10). Relative percentages correspond to the proportions of untreated (100%) cells. Detection of both total protein (stain-free blot) and detected bands was performed using the Chemidoc^TM^MP Imaging System (Biorad), and normalization was performed using Image Lab Software (Biorad).

Lastly, the inflammasome effector protein pro-caspase-1’s gene and protein expression were assessed. The absence of ICP27 in the HSV-1ΔICP27-deleted viral vector caused a large rise in pro-caspase-1 RNA starting eight hours after infection, according to the real-time PCR results (Figure 5A). Pro-caspase-1 protein, as assessed at 10 h.p.i., also demonstrated a noteworthy rise in HSV-1ΔICP27-deleted vector-infected cells (919%) in contrast to HSV-1 w.t. virus (35%) (Figure 5B,C). These findings show that ICP27 in HSV-1 w.t. causes a decrease in the pro-caspase-1 gene and protein production that is comparable to or even lower than in untreated cells, indicating that ICP27 functions as an AIM 2 signalsome inhibitor. 

### 2.5. Inhibition of AIM 2 Inflammasome Constituent Proteins by ICP27 Results in Downregulation of Active Caspase-1

**Figure 6:**
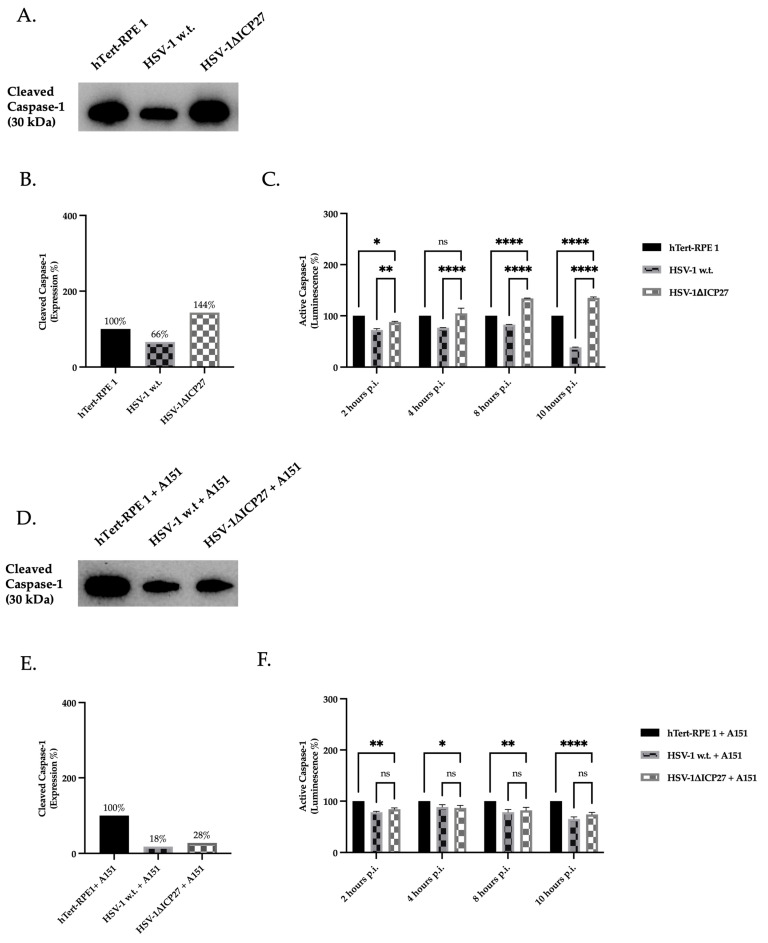
Protein expression and enzymatic activity of caspase-1. (**A**,**D**) Western blot assay for transient caspase-1 (30 kDa) in both untreated (**A**) and pre-treated with AIM 2 inhibitor A151 (**D**) hTert-RPE 1, HSV-1 w.t., and HSV-1ΔICP27-infected cells (M.O.I. of 3) at 10 h.p.i. (**B**,**E**) Densitometric quantification of caspase-1 levels was performed using stain-free blotting, and the results were reported as a percentage calculated from the ratio of normalized protein levels to total protein (Supplementary Materials–Figures S11–S14). Relative percentages correspond to the proportions of untreated (100%) cells. Detection of both total protein (stain-free blot) and detected bands was performed using the Chemidoc^TM^MP Imaging System (Biorad) and normalization was performed using Image Lab Software (Biorad). (**C**,**F**) Bioluminescent assay for active caspase-1 in untreated (**C**) and pre-treated with AIM 2 inhibitor A151 (**F**) hTert-RPE 1, HSV-1 w.t., and HSV-1ΔICP27-infected cells (M.O.I. of 3) at 2, 4, 8, and 10 h.p.i. Values are average SEM ± from three biological replicates. Two-way ANOVA was performed. ns *p* ≥ 0.1, *, *p* < 0.1, **, *p* < 0.01, ****, *p* < 0.0001.

To assess whether the reduction in gene and protein expression of inflammasome constituent proteins caused by ICP27 had an effect on the conversion of pro-caspase-1 to caspase-1, the protein expression of the active caspase-1 enzyme was evaluated. Therefore, the protein expression of active caspase-1 (p20 caspase-1; 20 kDa) was quantified in untreated hTert-RPE 1 cells, and infected cells with HSV-1 w.t. and HSV-1ΔICP27 virus (Figure 6A,B). Contrary to our expectations, the identified caspase-1 has a molecular weight of 30 kDa, which is surprising. Indeed, Boucher et al. showed that active p20 caspase-1 is extremely unstable once produced: self-cleavage leading to the removal of the card domains of the p30 dimer results in the release of p20, which, in turn, triggers the termination of activity. In contrast, the active transient p30 species remains bound to the inflammasome, making the holoenzyme active, since caspase-1 requires an association with the complex to maintain its activity [53]. The results show that the presence of the *ICP27* gene in cells infected with HSV-1 w.t. determines the reduction in p30 levels compared to untreated cells. In contrast, the absence of the *ICP27* gene in cells infected with HSV-1ΔICP27 results in an increase (143.8%) compared to both untreated (100%) and HSV-1 infected cells (65.8%). To assess the activity of caspase-1, a bioluminescent assay was performed (Figure 6C): the assay was conducted in parallel by treating the cellular samples with or without a selective caspase-1 inhibitor (Ac-YVAD-CHO) to selectively measure caspase-1 activity. From the data obtained by analyzing untreated cells and cells infected with HSV-1 w.t. or the HSV-1ΔICP27 mutant virus, a reduction in enzyme activity was observed in cells infected with HSV-1 w.t. expressing the ICP27 protein compared to untreated cells. In contrast, a significant increase in caspase-1 enzyme activity was observed in cells infected with the HSV-1ΔICP27 mutant vector from 8 h.p.i. compared with both untreated cells and cells infected with HSV-1 w.t. To see whether the effect of ICP27 on caspase-1 could depend on the AIM 2 sensor protein, we evaluated both caspase-1’s expression (Figure 6D,E) and activity (Figure 6F) by treating cells with the AIM 2 inhibitor ODN TTAGGG (A151) prior to infection. The results showed that both caspase-1 enzyme expression and activity in pre-treated cells with A151 and subsequently infected with HSV-1 w.t. or HSV-1ΔICP27 virus are comparable, and in both cases were lower than the enzyme activity of untreated cells. In detail, the protein expression of transient caspase (p30) in HSV-1 w.t. and HSV-1ΔICP27-infected cells was 18% and 28% compared to untreated cells (100%), respectively. In conclusion, the presence of the *ICP27* gene in HSV-1 w.t. resulted in a reduction in the enzymatic activity of the caspase-1 enzyme. This decrease is associated with the suppression of the AIM 2 inflammasome by ICP27. When pre-treated with an AIM 2 inhibitor, the expression and activity of active caspase-1 in cells infected with HSV-1 w.t. and HSV-1ΔICP27 are equivalent. In summary, this may indicate a close relationship between the sensor protein AIM 2 and the inflammasome inhibition induced by the ICP27 protein. 

### 2.6. The Inhibition of Caspase-1 by ICP27 Affects the Expression and Release of Pro-Inflammatory Cytokines IL-1β and IL-18 

**Figure 7:**
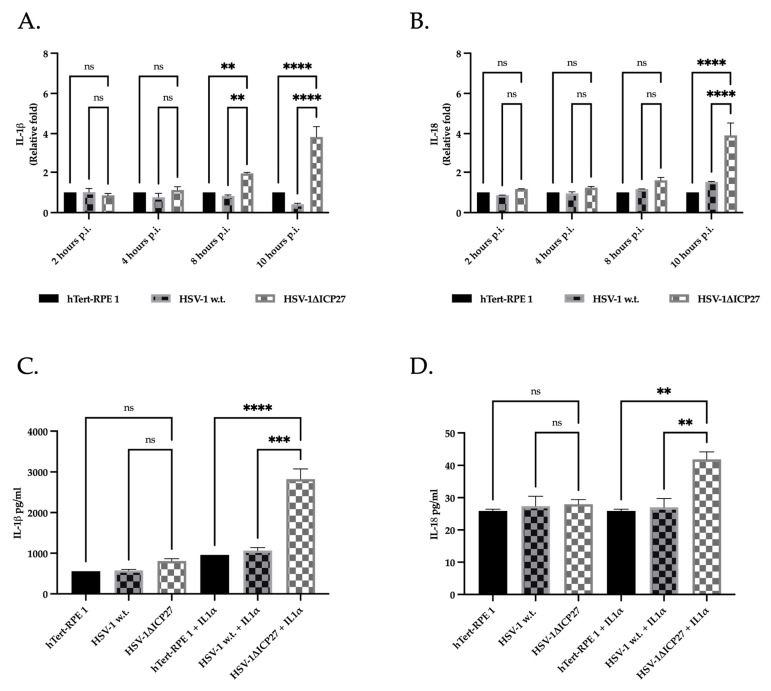
Gene expression and quantification of released IL-1β and IL-18. (**A**,**B**) Real-time PCR of untreated hTert-RPE 1, HSV-1 w.t., and HSV-1ΔICP27-infected cells (M.O.I. of 3) at 2, 4, 8, and 10 h.p.i for IL-1β (**A**) and IL-18 (**B**). Relative fold changes are related to the levels in untreated cells (hTert-RPE 1). Values are averages SEM ± from three biological replicates. Two-way ANOVA was performed. ns *p* ≥ 0.1, **, *p* < 0.01, ****, *p* < 0.0001. (**C**) ELISA assay for IL-1β in both untreated or stimulated with IL-1α hTert-RPE 1, HSV-1 w.t., and HSV-1ΔICP27-infected cells (M.O.I. of 3) at 10 h.p.i. Values are average SEM ± from three biological replicates. Two-way ANOVA was performed. ns *p* ≥ 0.1, ***, *p* < 0.001. ****, *p* < 0.0001. (**D**) ELISA assay for IL-18 in both untreated or stimulated with IL-1α hTert-RPE 1, HSV-1 w.t., and HSV-1ΔICP27-infected cells (M.O.I. of 3) at 10 h.p.i. Values are average SEM ± from three biological replicates. Two-way ANOVA was performed. ns *p* ≥ 0.1, **, *p* < 0.01.

We assessed the effect of ICP27 on the inflammasome pathway by measuring the expression of the IL-1β and IL-18 genes. The results shown in Figure 7A indicate that the relative fold change of IL-1β decreases from 2 h.p.i. in cells infected with HSV-1 w.t., in contrast to cells infected with the virus lacking the *ICP27* gene (HSV-1ΔICP27), where the relative fold change of IL-1β increases to be significantly greater starting from 8 h.p.i. The relative fold change of IL-18 (Figure 7B) in cells infected with HSV-1ΔICP27 also seems to rise gradually with infection progression, peaking at 10 h.p.i. In contrast to interleukin 1β, it has been reported that the relative fold change of IL-18 is higher in HSV-1-infected cells when compared to untreated cells.

Next, IL-1β and IL-18 released in the supernatant of untreated or HSV-1-infected cells expressing, or not, the *ICP27* gene were quantified at 10 h post-infection by performing an ELISA assay. The data show a slight but still significant increase in IL-1β in cells infected with the virus lacking the *ICP27* gene compared with both untreated cells and cells infected with HSV-1 w.t. (Figure 7C). In contrast, no difference was noted in the amount of IL-18 present in untreated cells and infected with HSV-1 w.t. or HSV-1ΔICP27 (Figure 7D). However, it has been verified that several viruses, including HSV, require a stronger inflammatory cell phenotype to induce interleukin production and release. Therefore, hTert-RPE 1 cells were incubated with interleukin 1α (IL-1α) before infection to induce a more pro-inflammatory cell type [54,55]. The results obtained (Figure 7C,D) show a marked increase in the basal levels of interleukins detected in all samples and a significant difference between the interleukins present in HSV-1 w.t. and HSV-1ΔICP27-infected cells. In fact, the amount of IL-1β detected in cells pre-stimulated with IL-1α and subsequently infected with HSV-1ΔICP27 was three-fold higher than in both untreated and HSV-1-infected cells. IL-18 was also found to be significantly higher in cells infected with HSV-1ΔICP27, although to a much lesser extent than IL-1β. 

## 3. Discussion

A viral pathogen’s infection of a host cell sets off a multilevel antiviral response; however, viruses have co-evolved with their hosts, resulting in a variety of mechanisms that enable them to overcome different innate immune response pathways and, consequently, adaptive immune responses [56,57,58]. Different strategies are used by some viruses, such as HSV-1, to counteract the induction of pro-inflammatory cytokines. Our understanding of the strategies used by HSV-1 to evade the responses activated by the infected cell has improved with the discovery of inflammasomes as innate immune signaling complexes [59,60,61]. Indeed, the development of more effective treatments for viral infection depends on a deeper comprehension of the roles that particular HSV-1 genes play and the immune system’s reaction to viral infection [62].

Therefore, we concentrated on researching how the HSV-1 gene ICP27 affects hTert-RPE 1 cells’ AIM 2 inflammasome pathway. Not only are hTert-RPE 1 cells target cells during primary HSV-1 infection at the ocular level, but they also modulate the immune response by releasing a variety of cytokines that affect the inflammatory response of neighboring immune cells, including macrophages, dendritic cells, and NK cells, which are located close to the retinal epithelial barrier [63,64,65,66].

ICP27 is an essential and multifunctional protein that can act on multiple levels with the objective of regulating cellular homeostasis in favor of the virus [42]. These multiple roles inevitably impact the innate immune response induced by the infected cell: in fact, it has been shown that ICP27 can interfere with antiviral cellular responses and immune signaling by counteracting cytokine induction through an interaction with various cellular systems: specifically, ICP27 targets NF-kB and STAT signaling pathways, thereby interfering with the transcription of *ISG*s genes (interferon-stimulated genes) [49,50,51,52].

**Figure 8:**
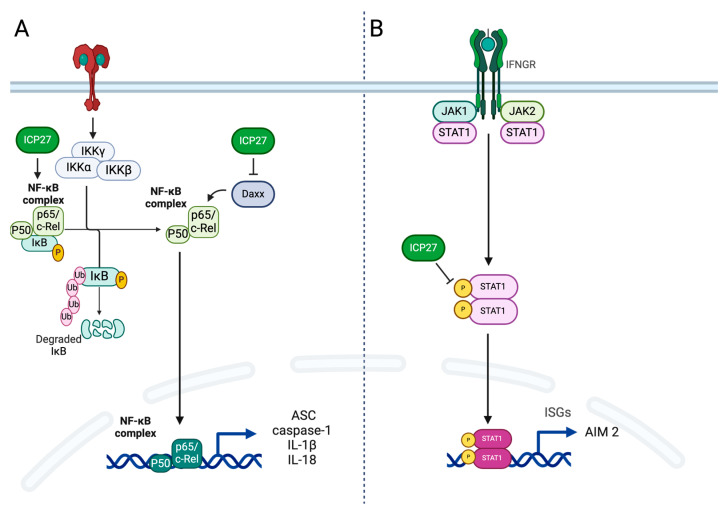
Role of ICP27 protein in immune evasion: (**A**) ICP27 is required for the inhibition of NF-kB by stabilizing the NF-kB inhibitor IkB and by repressing the sumoylation of Daxx factor that consists of the inhibition of the transcriptional activity of NfkB essential for transcription of ASC, caspase-1, IL-1β, and Il-18 genes. (**B**) ICP27 downregulates STAT1 phosphorylation and prevents the accumulation of STAT1 in the nucleus, which prevents transcription of the AIM 2 gene.

Our results show that ICP27 interferes by downregulating the gene and protein expression of heptosome constituents and, consequently, the release of pro-inflammatory cytokines. Specifically, our data indicate a decrease in the relative fold of the RNA of AIM 2, ASC, caspase-1, and interleukins 1β and 18 in HSV-1 infected cells, in which ICP27 is actively produced, compared to cells infected with HSV-1ΔICP27. Since the genes encoding the proteins that constitute the inflammasome are largely mediated by the transcriptional factor NF-kB, we have reason to believe that the ICP27 reduces the gene expression of inflammasome components through the inhibition of NF-kB transcriptional activity [67,68,69]. Although transcription of the gene encoding AIM 2 does not appear to be mediated by NF-kB, it has been observed that its transcription is dynamically controlled by various factors including STAT1, a transcriptional factor whose transition to the nucleus is downregulated by ICP27 [70,71]. Therefore, the role of ICP27 on the NF-kB and Jak/STAT signaling pathways is likely related to the downregulation of inflammasome constituent genes shown in our work (Figure 8). Moreover, the difference seen in gene expression between viruses expressing or not expressing ICP27 also translates into a difference in terms of protein expression: at 10 h post-infection, the quantification of proteins constituting the AIM 2 inflammasome, such as AIM 2, ASC, and pro-caspase-1, are found to be consistently lower in cells infected with HSV-1 w.t. than in the virus lacking *ICP27* gene. The reduced gene and protein expression of each component of AIM 2 inflammasome induced by the ICP27 protein induces a significant reduction in the activity of the heptosome, which is no longer able to efficiently catalyze the activation of pro-caspase-1 into its active form caspase-1. 

Finally, the inhibitory effect on the AIM 2 inflammasome pathway attributed to ICP27 results in a reduced downstream release of interleukins IL-1β and IL-18 processed by active caspase-1. IL-1β and IL-18 are crucial in the activation of the specific immune response: their production and release cause the release of an additional subset of cytokines from tissue cells by directing differentiation through stimulation of Th1, Th2, and Th17 populations. In this context, multiple studies suggest how IL-1β and IL-18 can act as a bridge between the innate and acquired immune response [72,73,74]. They can activate lymphocytes and increase T- and B-cell proliferation, stimulate natural killer cell activity and cytokine secretion, and contribute to T-cell proliferation, memory T-cell expansion, and dendritic cells. This underscores the importance of inflammasome activation in vaccine development and suggests novel approaches for optimizing vaccine formulation. When designing vaccines, activating inflammasomes can mimic the natural immune response to infection, leading to a more robust and effective immune reaction. Moreover, the maturation and activation of antigen-presenting cells, such as dendritic cells, which are essential for triggering adaptive immune responses, can also be facilitated by activating inflammasomes [75,76,77,78,79]. This can lead to a better antigen presentation and the activation of T cells, further enhancing the effectiveness of the vaccine [80]. 

In the case of herpetic viral vectors for vaccine purposes, targeting inflammasomes could improve the immune response against the virus. Herpes viruses, including HSV, possess immune evasion strategies that inhibit or evade the host’s immune response. By exploiting the innate immune pathways mediated by inflammasomes, vaccines can potentially overcome these evasion mechanisms and stimulate a more potent and durable immune response [81,82]. Therefore, focusing on the molecular mechanisms involved in the activation of inflammasomes could be useful to further advance the design of effective herpetic viral vectors used for vaccine purposes.

In conclusion, our work attempts to shed light on the role of the HSV-1 ICP27 protein in immune evasion through the inhibition of the AIM 2 inflammasome, improving the multiple known mechanisms that the virus presents to evade the innate immune response and adding useful information for more consciously developing effective vaccine strategies to counter herpetic infection.

## 4. Materials and Methods

### 4.1. Cell Lines

Vero (African green monkey epithelial cells, ATCC CCL-81) and 7b cells (Vero cells expressing the precocious genes coding for the ICP4 and ICP27 proteins of HSV-1 as previously described) were used in the preparation of viral stocks. Both Vero and 7b cells were grown in Dulbecco’s minimal essential medium DMEM containing 100 U/mL penicillin-100 μg/mL streptomycin, 2 mM L-glutamine, and 10% heat-inactivated Fetal Bovine Serum (All from Euroclone, Milano, Italy). 7b cells require the addition of 100 μg/mL of antibiotic G418 (Geneticin solution; Euroclone) to maintain selection [83].

hTert-immortalized retinal pigment epithelial cells (ATCC, Rockville, MD, USA; CRL-4000) were cultured in Dulbecco’s modified Eagle’s medium (DMEM)/F12 medium supplemented with 10% heat-inactivated fetal bovine serum (FBS), 2 mM L-glutamine, 100 U/mL penicillin, 100 μg/mL streptomycin (All from Euroclone), and 0.01 mg/mL hygromycin B (Merck Life Science S.r.l., Milano, Italy). 

Vero, 7b, and hTert-RPE 1 were grown in monolayers and were kept at 37 °C in complete sterility with an atmospheric CO_2_ content of 5% and passaged at a ratio of 1:5 to 1:10 twice weekly using trypsin-EDTA solution 1X (Merck Life Science S.r.l., Milano, Italy). 

### 4.2. Plasmid Construction

To construct the herpetic vector HSV-1ΔICP27 used in this project, the commercial plasmid pBSSK (Stratagene, San Diego, CA, USA) was used for subsequent cloning. In detail, the 2421 base pair (bp) of BamHI-SacI fragment from the HSV-1 genome (nucleotides 113323 to 115743) containing *UL54*, corresponding to the entire ICP27 sequence, was cloned into the BamHI-SacI sites of pBSSK plasmid, resulting in the pBICP27 plasmid.

### 4.3. Viruses Construction

The wild-type strain used in the study was HSV-1 strain F (NCBI:txid10304). To evaluate the effect of the *ICP27* gene of HSV-1 on the inflammatory pathway, the activity of HSV-1 w.t. was compared with HSV-1ΔICP27 mutant virus, in which the HSV-1 (F) virus was deleted from the ICP27 gene. The HSV-1ΔICP27 vectors were generated from a pre-existing BAC-HSV-1 (Bacterial Artificial Chromosome containing a full-length infectious clone of HSV-1 strain F), with the BAC vector inserted into the intergenic region between *UL3* and *UL4* of HSV-1 genome kindly provided by C. Fraefel. HSV-1ΔICP27 recombinant vector was obtained using the “recombineering” system in bacteria SW102 previously described [84,85,86,87]. To evaluate the absence/presence of ICP27 Western blot analyses were performed. 

### 4.4. Viruses: Propagation and In Vitro Titration

Vero cells were infected with M.O.I. of 0.01 with HSV-1 strain F and 7b cells were infected with M.O.I. of 0.03 HSV-1ΔICP27, according to the standard procedure [88]. Briefly, when 100% of cytopathic effect was observed, cells were harvested and spun down at 1500 rpm for 10 min to collect cellular pellets and the supernatant was further centrifuged at 19,000 rpm for 30 min to collect viral pellets. Cell pellets were then subjected to 3 cycles of freeze/thaw, sonicated, and centrifuged at 3000 rpm for 10 min to discharge cellular debris. The supernatant collected after this centrifugation was mixed with the viral pellet previously obtained and the mix was further centrifuged at 19,000 rpm for 30 min. Final viral pellets were resuspended in phosphate-buffered saline (PBS), sonicated, aliquoted, and stored at −80 °C until use. To determine the viral titer (i.e., p.f.u/mL), the viral stock was serially diluted 1:10 in 1 mL of culture medium DMEM High-Glucose with 10% FBS. Each dilution was added to monolayer cultures of Vero or 7b cells previously seeded (1 × 10^6^ cells/well) in six-well plates for 1 h at 37 °C. After incubation, the medium was replaced with fresh medium containing 1% methylcellulose (Merck Life Science S.r.l., Milano, Italy). A total of 72 h later, viral titer was determined using the plaque assay method [88,89]. Cells were washed twice with Tris-buffered saline (TBS: 50 mM Tris, 150 mM NaCl, pH 7.6), fixed and stained with a solution of 50% methanol in water containing 1% of crystal violet for 30 min at room temperature (all the chemical reagents and diluents have been purchased from Merck Life Science S.r.l., Milano, Italy)The viral titer was determined by multiplying the number of the plaques by the dilution factor. 

### 4.5. Transfection

In order to verify the expression of ICP27 protein, transfection of hTert-RPE 1 with pBBSK and pBICP7 was performed. Confluent hTert-RPE 1 monolayers in six-well plates were transfected with 3 μg of pICP27 plasmid or its control pBSSK for 24 h with Lipofectamine 2000, as described by the manufacturer (Invitrogen Waltham, MA, USA).

### 4.6. Infection

To evaluate the role of ICP27 on AIM 2 inflammasome pathway, hTert-RPE 1 cells were infected with the HSV-1 w.t. strain F or HSV-1ΔICP27 mutant virus at 3 M.O.I. Viral particles were diluted in culture medium and added to cell monolayers (1 × 10^6^ cells/6-well plates or 2 × 10^4^ cells/96-well plates) for 2, 4, 8, and 10 h at 37 °C. After 2, 4, 8, and 10 h.p.i., infected cells were collected and processed for PCR, Western blot, ELISA, and bioluminescent analyses, as described below. Untreated hTert-RPE 1 cells were used as negative control. 

### 4.7. AIM 2 Inhibition

To inhibit AIM 2 protein sensor, 50 µM of ODNTTAGGG (A151) (tlrl-ttag151, InvivoGen; Roma, Italy) was added to the culture medium of hTert-RPE 1 (2 × 10^5^/well) for 1 h at 37 °C. After incubation with AIM 2 inhibitor, cells were infected with the HSV-1 w.t. strain F or HSV-1ΔICP27 mutant virus at 3 M.O.I. for 2, 4, 8, and 10 h at 37 °C. Negative control was represented by uninfected cells. 

### 4.8. RNA Extraction and Real-Time PCR

After infection, cells were collected by scraping and low-speed centrifugation and resuspended in TRI-Reagent (Merck Life Science S.r.l., Milano, Italy). The integrity of RNA was checked on 1% agarose gels and quantified at 260/280 ratio by using Eppendorf BioSpectometer basic (Eppendorf srl; Milano, Italy). RNA was converted to cDNA using High-Capacity cDNA Reverse Transcription Kit according to the production protocol (Applied Biosystems; Monza, Italy). Dilution of cDNA was performed to obtain a uniform amount of cDNA in all samples. Real-time PCR was performed by using CFX96 Touch Real-time PCR Detection System (Biorad; Italy): all samples (unknown, standard, and non-template control (NTC)) were run in triplicate according to the desired specifications of PowerUp SYBR Green Master Mix for qPCR (Applied Biosystems; Italy) used. Subsequently, Ct (cycle threshold) of the various samples was analyzed: relative quantification of target genes expression was performed by using 2^−ΔΔCT^ method in order to normalize the endogenous reference genes relative to the untreated control. Melting curves of all real-time PCR end products were analyzed to determine authentic products and contamination of non-specific products and primer dimer. GAPDH and 18s were used as internal controls. Target and endogenous genes are listed in Table 1 [90].

### 4.9. Protein Extraction and Western Blot Assay

Infected or untreated cells were rinsed once with PBS, collected using scraping and low-speed centrifugation, resuspended in lysis buffer (50 mM Tris-HCl pH 8, 1% Triton X-100, 0.5% NP-40, 10 mM β-mercaptoethanol, 4% glycerol (all the chemical reagents and diluents have been purchased from Merck Life Science S.r.l., Milano, Italy) and Protease inhibitor mix (Roche; Monza Italy), and boiled for 10 min. Samples were boiled for 10 min at 100 °C, frozen for 2 min, and sonicated (2 times × 30 s). Subsequently, they were centrifuged at 14,000 rpm for 10 min, and the supernatant was quantified using the BCA protein Assay kit (Fisher Scientific Italia, Segrate, MI, Italy). Specifically, protein quantification was performed using the colorimetric BCA protein assay. Samples containing the previously extracted proteins were treated with bicinchoninic acid (BCA) so that the proteins and peptides reduced the Cu^2+^ ions to Cu^1+^ ions in basic environment. The Cu^1+^ ions react with the acid to form a purple complex (Cu^1+^ ion chelated by two BCA molecules). The color was measured at a wavelength of 562 nm using the Glomax Discover microplate reader instrument (Promega; Milano Italy). The unknown protein concentration was extrapolated by referring to the absorbance relative to a protein of known concentration: bovine serum albumin (BSA). Total proteins (30 μg) of each sample were run on TGX Stain-Free FastCast Acrylamide Kit 10% (Biorad, Segrate, MI, Italy) and proteins were transferred to nitrocellulose membranes (Trans-Blot Turbo, RTA Transfer Kit, Nitrocellulose; Biorad; Segrate, MI, Italy). Filters were then blocked with TBST at pH 7.4 (NaCl 8 gr, KCl 0.2 gr, Tris-Base 3 gr, and 0.05% of Tween 20 for 1 L of water solution) for 1 h at room temperature. The antibodies were diluted in a working solution composed of TBST with 5% Nonfat-Dried Milk bovine (Merck Life Science S.r.l., Milano, Italy). Filters were incubated with primary antibody for 1 h at room temperature (Table 2). After washing with TBST, filters were incubated with the goat anti-mouse or anti-rabbit IgG horseradish peroxidase-linked secondary antibodies (Table 2). Immunocomplexes were detected using ECL detection kit NeoPRO Femto (NeoBiotech; Nanterre, France) and the quantification of the detected bands was related to the total proteins transferred to the membrane (stain-free blot). Quantification of both total protein (Stain-free blot) and detected bands was performed using the Chemidoc^TM^MP Imaging System (Biorad, Segrate, MI, Italy) and normalization was performed using Image Lab Software (v.6.0.0, Biorad, Segrate, MI, Italy) [91].

### 4.10. Caspase-1 Bioluminescent Inflammasome Assay 

The assay used to assess caspase-1 activity and, thus, inflammasome activation, was the bioluminescent Caspase-Glo 1 Inflammasome Enzyme Assay (Promega, Milano, Italy). A total of 5 × 10^5^ hTert-RPE 1 cells/well were infected with HSV-1 w.t. virus or HSV-1ΔICP27 mutant virus at 3 M.O.I. Uninfected cells were used as negative controls. Active caspase-1 activity was quantified at 2, 4, 8, and 10 h.p.i. with the GloMax luminometer. All measurements were performed following the manufacturer’s protocols. The working principle of this assay involves the use of a luminogenic caspase-1 enzyme substrate (Z-WHED-AMINOLUCIFERIN) included in a lytic reagent optimized for caspase-1 and luciferase activity. Thus, the treatment of the samples at the time mentioned above involves lysis, allowing the binding of the Z-WEHD-aminoluciferin substrate with caspase-1 and the subsequent cutting of aminoluciferin using Z-WEHD. Simultaneous to the caspase-1 cutting reaction, the cleavage of amino-luciferin by the enzyme luciferase (Ultra-Glo™ Recombinant Luciferase) present in the reagent occurs, resulting in the release of the light signal, which is proportional to caspase-1 activity [92].

To avoid non-specific substrate cuts by caspase-1, it was necessary to add a selective proteasome inhibitor, MG-132, at a final concentration of 60 µM. In addition, to ensure specificity of the cutting by the substrate caspase-1, we performed the assay in parallel for each sample with the addition of a caspase-1-specific inhibitor, Ac-YVAD-CHO, at a final concentration of 1 µM, which does not, however, inhibit any of the other caspases that cross-react with caspase-1 (e.g., caspases 4 and 5). In parallel, all samples were treated with the specific inhibitor of AIM 2 ODN TTAGGG (A151) (tlrl-ttag151, InvivoGen, Italy) to verify the inhibition exerted by the ICP27 gene of HSV-1 by comparing the caspase-1 activity in both cells infected with HSV-1 w.t. or with HSV-1ΔICP27 mutant virus.

### 4.11. ELISA and IL-1α Treatment

After 10 h of infection with HSV-1 w.t. virus or HSV-1ΔICP27 mutant virus, supernatants of infected hTert-RPE 1 were collected and centrifuged at 3000 rpm for 20 min to remove cell debris. IL-1β and IL-18 were quantified using the Human IL-1β Elisa kit and Human IL-18 Elisa kit, respectively (E0143Hu and E0147Hu, BTLabs, Shanghai Korain, China). In order to generate a pro-inflammatory status of hTert-RPE, cells were pretreated with 4 ng/mL of IL-1α (Immunotools, Friesoythe; Germany) for 48 h before the infection. All measurements were made following the manufacturer’s protocols.

### 4.12. Statistical Analysis

All tests were performed three times in triplicate, and the statistical analysis was performed using two-way or one-way ANOVA followed by Dunnett’s multiple comparisons test with GraphPad Prism version 9.0.0 for MacOS, GraphPad Software, San Diego, CA, USA, with *p* ≤ 0.05 to identify significant differences.

## Figures and Tables

**Table 1:** Primers used for real-time PCR analysis.

Gene	Primer Forward	Primer Reverse
Caspase 1	TGAATACCAAGAACTGCCCAAG	GCATCATCCTCAAACTCTTCTGTAG
ASC	CGCGAGGGTCACAAACGT	TGCTCATCCGTCAGGACCTT
AIM 2	ATCTCCTGCTGCCTTCTTGG	AAGTCTCTCCTCATGTTAAGCCTG
IL-1β	AGATGATAAGCCCACTCTACAG	ACATTCAGCACAGACTCTC
IL-18	CAGCCGCTTTAGCAGCCA	GCAAGGAATGTCTCCCAGTGC
GAPDH	GGTGTGAACCATGAGAAGTA	GAGTCCTTCCACGATACCAA
18s	GTAACCCGTTGAACCCCATT	CCATCCAATCGGTAGTAGCG

**Table 2:** Antibodies used for Western blot analysis.

Antibodies	Source	Identifier
Mouse monoclonal anti-ICP27 antibody	Virusys	Cat#P1113
Mouse monoclonal Human Caspase-1 Antibody	R&D System	Cat#MAB6215
Rabbit polyclonal anti-caspase 1 (c-term) antibody	Biorad	Cat#AHP963
Rabbit polyclonal anti-TMS1/ASC antibody	Abcam	Cat#EPR23978-28
Rabbit monoclonal anti-AIM2 antibody	R&D System	Cat#MAB9965
Polyclonal Goat Anti-Mouse Immunoglobulins/HRP	Dako	Cat#P0447
Polyclonal Goat Anti-Mouse Immunoglobulins/HRP	Dako	Cat#P0448

## Data Availability

Data are contained within the article and Supplementary Materials.

## Supplementary Materials

The following supporting information can be downloaded at: https://www.mdpi.com/article/10.3390/ijms25094608/s1.
