# Supplementary material for: Herpes Simplex Virus ICP27 Protein Inhibits AIM 2-Dependent Inflammasome Influencing Pro-Inflammatory Cytokines Release in Human Pigment Epithelial Cells (hTert-RPE 1)
Source: Int J Mol Sci. 2024 Apr 23;25(9):4608. doi: 10.3390/ijms25094608 (PMC11083950; doi:10.3390/ijms25094608)
Supplement: Supplementary file 1 [file ijms-25-04608-s001.zip › ijms-2953300-supplementary.pdf]

Figure S1

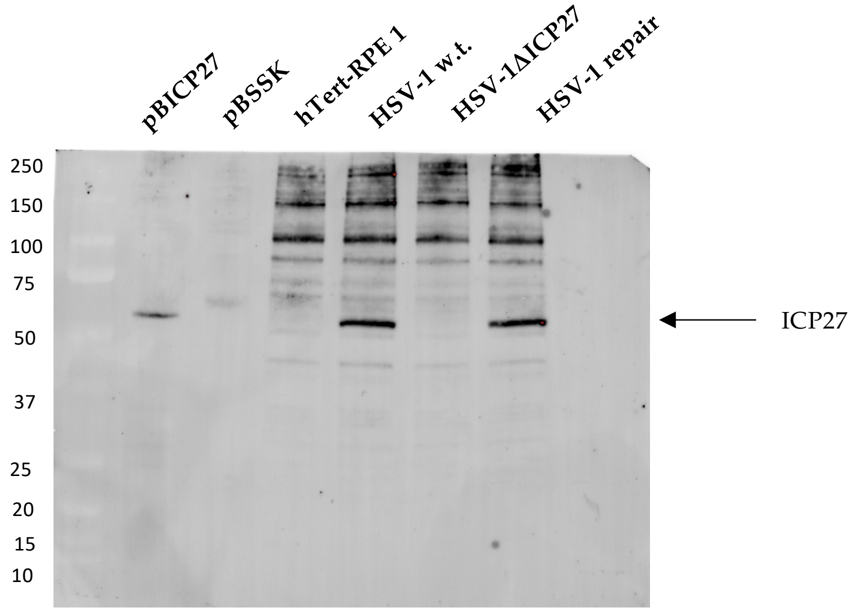

Figure S1: Figure 2 uncropped.

Western blot assay for ICP27 (63 kDa) in cells transfected with pBICP27 and pBSSK and in HSV-1 w.t., HSV-1ΔICP27 and HSV-1 repair infected cells at 24 hours post transfection or infection.

Marker:
Precision Plus Protein- All blue Prestained Protein standard (Biorad)

Figure S2

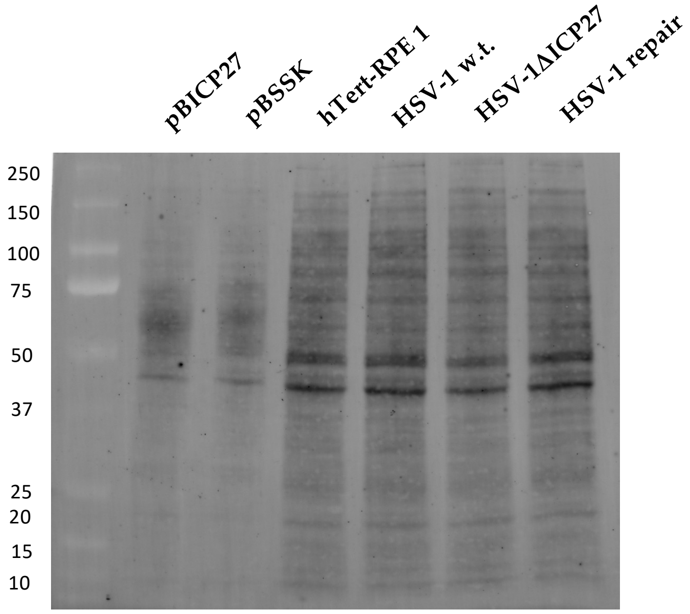

Figure S2: Total protein (Stain-free blot) of cells transfected with pBICP27 and pBSSK and infected with HSV-1 w.t., HSV-1ΔICP27 and HSV-1 repair at 24 hours post transfection or infection. Detection of total proteins was performed using the Chemidoc™MP Imaging System (Biorad). Normalization of detected bands [Figure 2] was performed using Lab Software (Biorad).

Figure S3

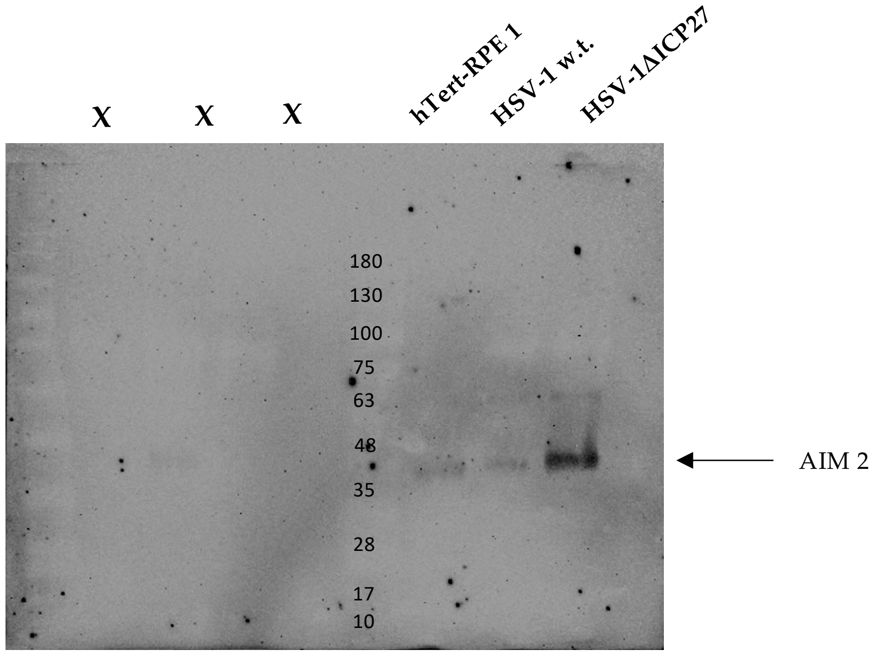

Figure S3: Figure 3B uncropped.

Western blot assay for AIM 2 (39 kDa) in untreated hTert-RPE 1, HSV-1 w.t. and HSV-1ΔICP27 infected cells (M.O.I. of 3) at 10 h.p.i.

Marker:
BlueStar Prestained Protein Marker (Nippon Genetics EUROPE GmbH)

Figure S4

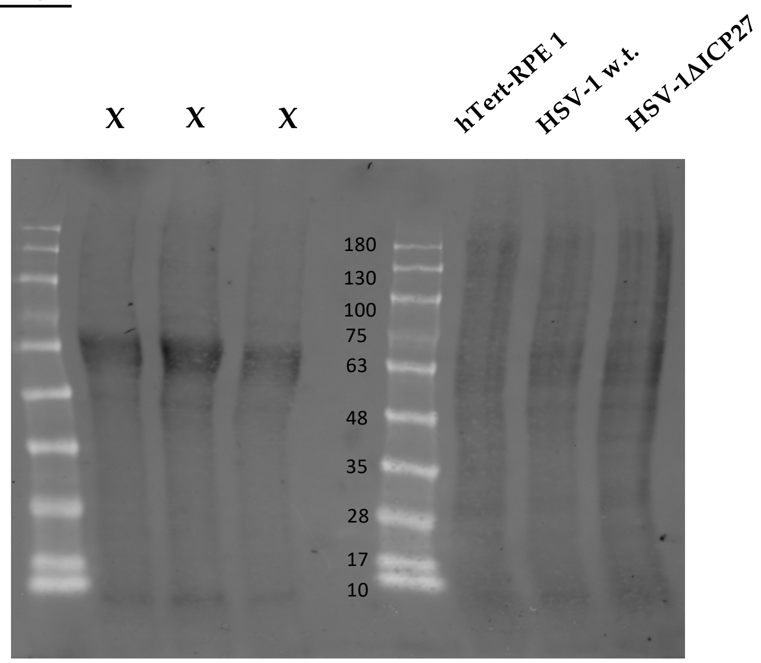

Figure S4: Total protein (Stain-free blot) of untreated hTert-RPE 1, HSV-1 w.t. and HSV-1ΔICP27 infected cells (M.O.I. of 3) at 10 h.p.i. Detection of total proteins was performed using the Chemidoc^{TM}MP Imaging System (Biorad). Normalization of detected bands [Figure 3B] was performed using Lab Software (Biorad).

Figure S5

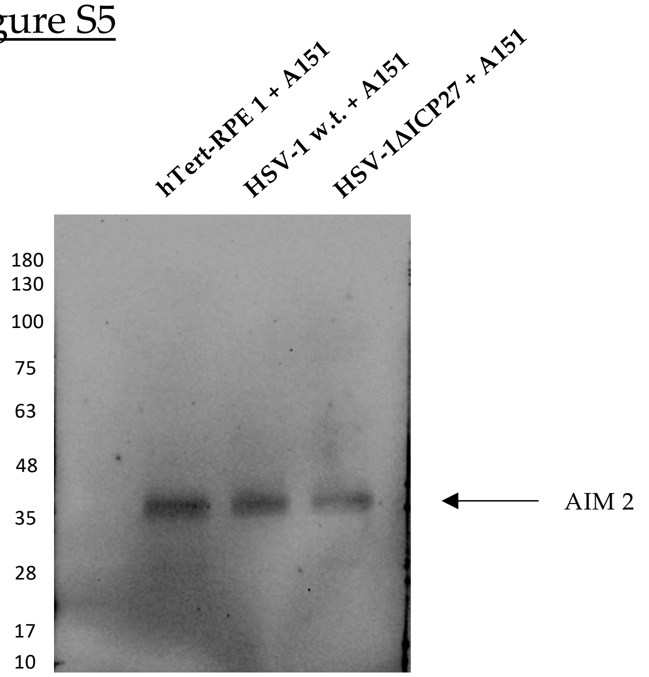

Figure S5: Figure 3D uncropped.

Western blot assay for AIM 2 (39 kDa) in AIM 2 inhibitor (A151) pre-treated hTert-RPE 1, HSV-1 w.t. and HSV-1ΔICP27 infected cells (M.O.I. of 3) at 10 h.p.i.

Marker:
BlueStar Prestained Protein Marker (Nippon Genetics EUROPE GmbH)

Figure S6

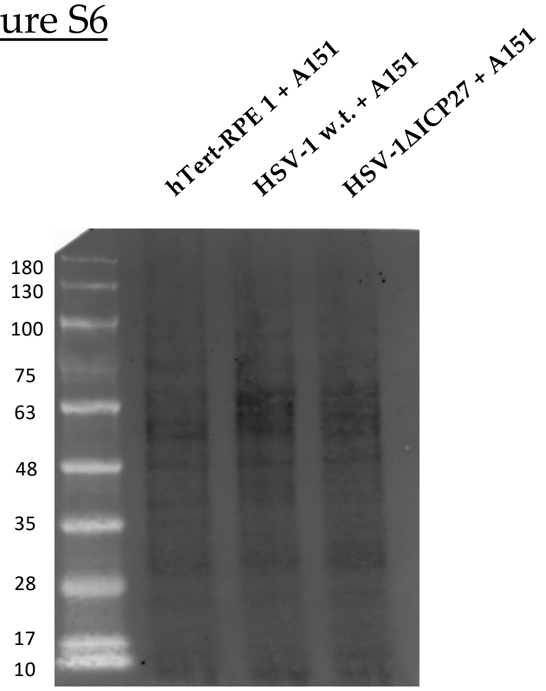

Figure S6: Total protein (Stain-free blot) of AIM 2 inhibitor (A151) pre-treated hTert-RPE 1, HSV-1 w.t. and HSV-1ΔICP27 infected cells (M.O.I. of 3) at 10 h.p.i. Detection of total proteins was performed using the Chemidoc^{TM}MP Imaging System (Biorad). Normalization of detected bands [Figure 3D] was performed using Lab Software (Biorad).

Figure S7

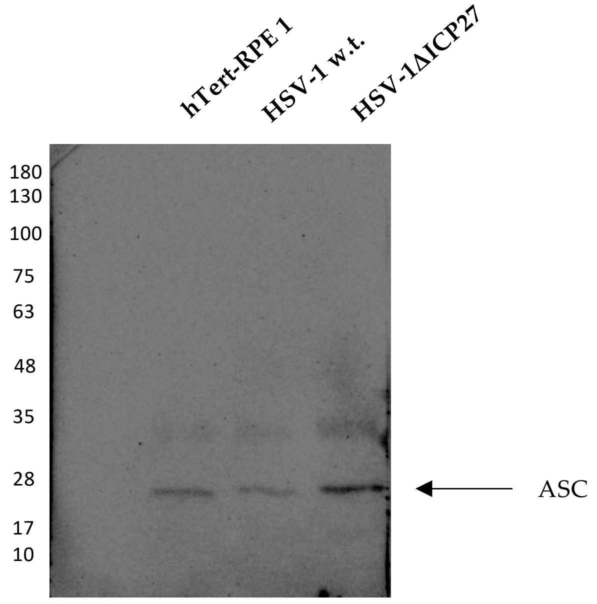

Figure S7: Figure 4B uncropped.

Western blot assay for ASC (22 kDa) in untreated hTert-RPE 1, HSV-1 w.t. and HSV-1ΔICP27 infected cells (M.O.I. of 3) at 10 h.p.i.

Marker:
BlueStar Prestained Protein Marker (Nippon Genetics EUROPE GmbH)

Figure S8

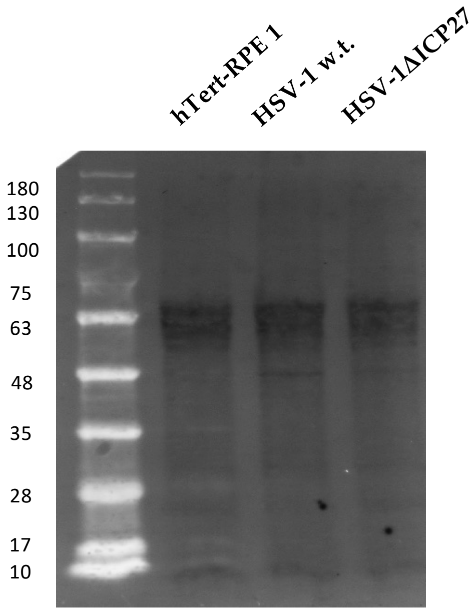

Figure S8: Total protein (Stain-free blot) of untreated hTert-RPE 1, HSV-1 w.t. and HSV-1ΔICP27 infected cells (M.O.I. of 3) at 10 h.p.i. Detection of total proteins was performed using the Chemidoc^{TM}MP Imaging System (Biorad). Normalization of detected bands [Figure 4B] was performed using Lab Software (Biorad).

Figure S9

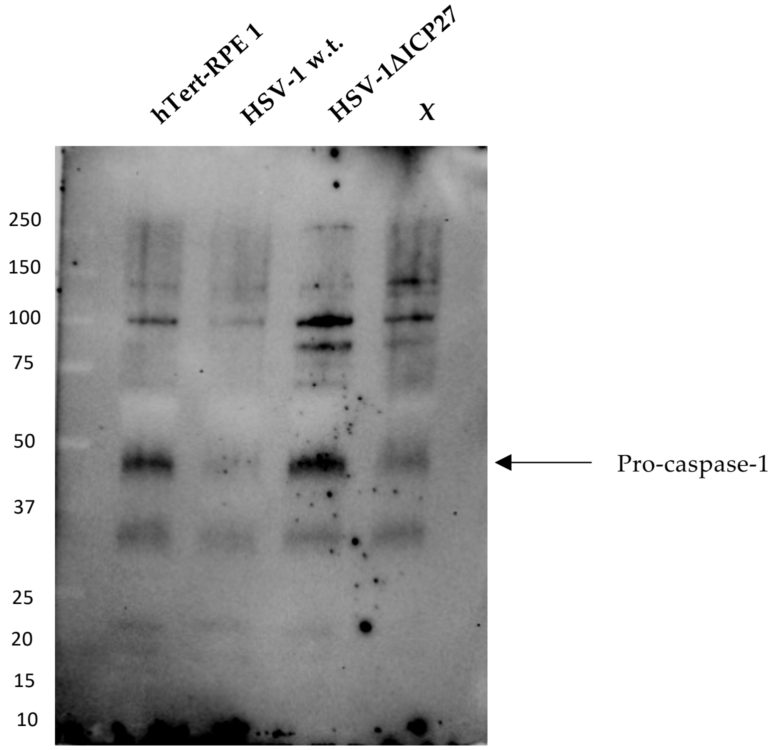

Figure S9: Figure 5B uncropped.

Western blot assay for pro-caspase-1 (45 kDa) in untreated hTert-RPE 1, HSV-1 w.t. and HSV-1ΔICP27 infected cells (M.O.I. of 3) at 10 h.p.i.

Marker:
Precision Plus Protein- All blue Prestained Protein standard (Biorad)

Figure S10

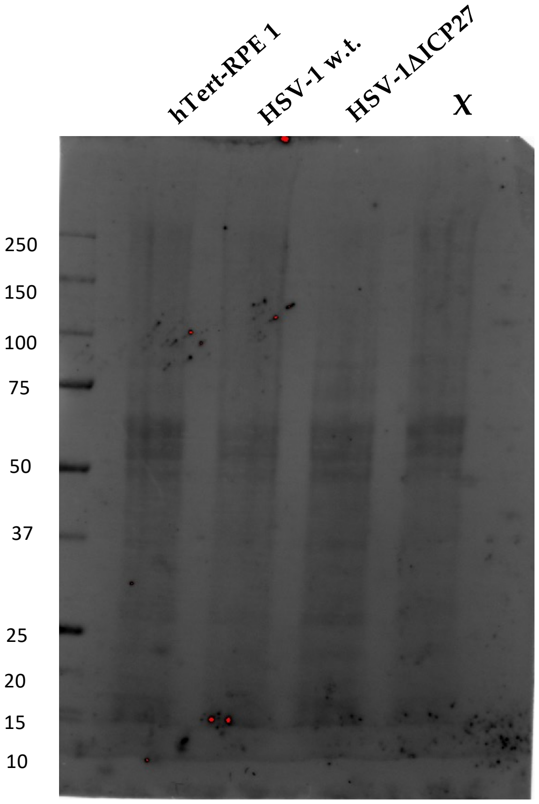

Figure S10: Total protein (Stain-free blot) of untreated hTert-RPE 1, HSV-1 w.t. and HSV-1ΔICP27 infected cells (M.O.I. of 3) at 10 h.p.i. Detection of total proteins was performed using the Chemidoc^{TM}MP Imaging System (Biorad). Normalization of detected bands [Figure 5B] was performed using Lab Software (Biorad).

Figure S11

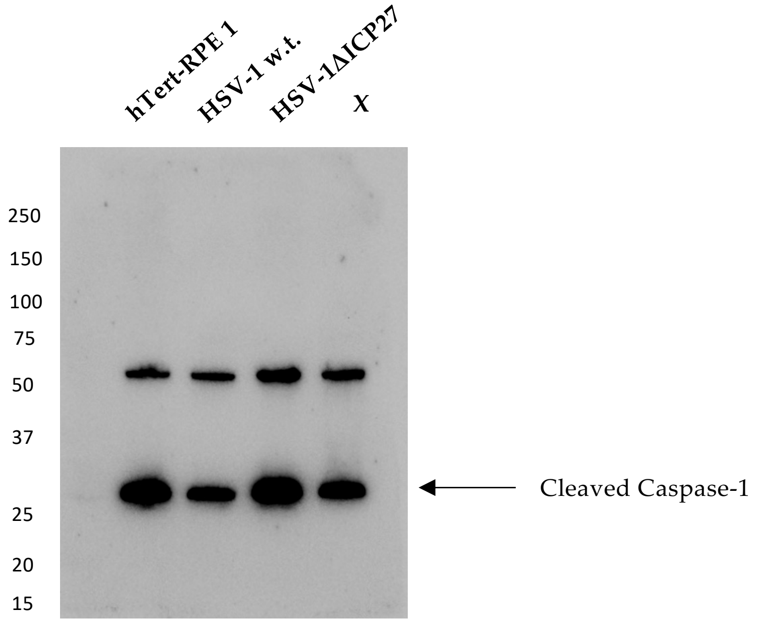

Figure S11: Figure 5B uncropped

Western blot assay for transient caspase (30 kDa) in untreated hTert-RPE 1, HSV-1 w.t. and HSV-1ΔICP27 infected cells (M.O.I. of 3) at 10 h.p.i.

Marker:
Precision Plus Protein- All blue Unstained Protein standard (Biorad)

Figure S12

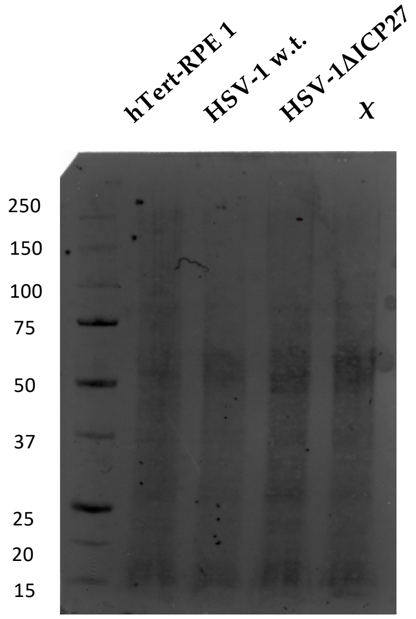

Figure S12: Total protein (Stain-free blot) of untreated hTert-RPE 1, HSV-1 w.t. and HSV-1ΔICP27 infected cells (M.O.I. of 3) at 10 h.p.i. Detection of total proteins was performed using the Chemidoc^{TM}MP Imaging System (Biorad). Normalization of detected bands [Figure 6A] was performed using Lab Software (Biorad).

Figure S13

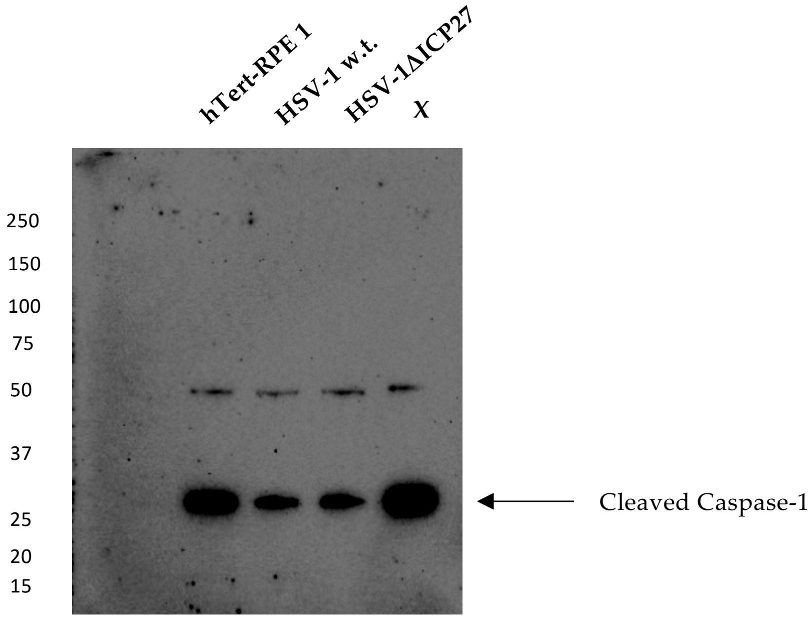

Figure S13: Figure 7 uncropped.

Western blot assay for transient caspase 1 (30kDa) in untreated hTert-RPE 1, HSV-1 w.t. and HSV-1ΔICP27 infected cells (M.O.I. of 3) at 10 h.p.i.

Marker:
Precision Plus Protein- All blue Unstained Protein standard (Biorad)

Figure S14

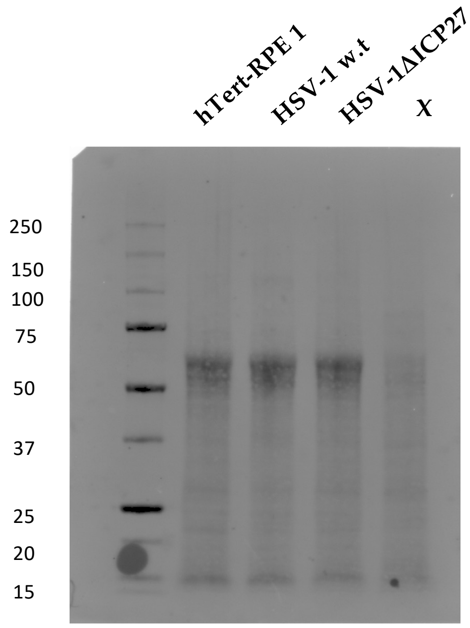

Figure S14: Total protein (Stain-free blot) of untreated hTert-RPE 1, HSV-1 w.t. and HSV-1ΔICP27 infected cells (M.O.I. of 3) at 10 h.p.i. Detection of total proteins was performed using the Chemidoc^{TM}MP Imaging System (Biorad). Normalization of detected bands [Figure 6D] was performed using Lab Software (Biorad).
